# Changes in plasma C1q, apelin and adropin concentrations in older adults after descending and ascending stair walking intervention

**DOI:** 10.1038/s41598-021-96631-x

**Published:** 2021-09-03

**Authors:** Trevor C. Chen, Tsang-Hai Huang, Wei-Chin Tseng, Kuo-Wei Tseng, Chung-Chan Hsieh, Mei-Yen Chen, Tai-Ying Chou, Yuh-Chuan Huang, Hsin-Lian Chen, Kazunori Nosaka

**Affiliations:** 1 Department of Physical Education and Sport Sciences, National Taiwan Normal University, P.O. Box 97-71, Wenshan Wansheng, Taipei City, 11699 Taiwan; 2 Institute of Physical Education, Health and Recreation, National Cheng-Kung University, Tainan City, Taiwan; 3 Department of Exercise and Health Sciences, University of Taipei, Taipei City, Taiwan; 4 Graduate Institute of Sport, Leisure and Hospitality Management, National Taiwan Normal University, Taipei City, Taiwan; 5 Department of Athletic Performance, National Taiwan Normal University, Taipei City, Taiwan; 6 Physical Education Office, Ming Chuan University, Taipei City, Taiwan; 7 Department of Physical Education, Health and Recreation, National Chiayi University, Chaiyi County, Taiwan; 8 Centre for Exercise and Sports Science Research, School of Medical and Health Sciences, Edith Cowan University, Joondalup, WA Australia

**Keywords:** Ageing, Physiology

## Abstract

This study compared changes in plasma complement component 1q (C1q), apelin and adropin concentrations in older obese women after descending (DSW) and ascending stair walking (ASW) training (n = 15/group) performed twice a week for 12 weeks, with gradual increases in exercise time from 5 to 60 min. Fasting blood samples were collected 3 days before the first and 4 days after the last training session. The improvements in the maximal voluntary isometric contraction (MVIC) strength of the knee extensors, functional physical fitness [e.g., 30-s chair stand (CS) performance], resting systolic blood pressure (SBP), insulin sensitivity [e.g., oral glucose tolerance test (OGTT)] and blood lipid profiles [e.g., total cholesterol (TC)] were greater (*p* < 0.05) in the DSW than ASW group. Plasma C1q decreased (− 51 ± 30%), and apelin (23 ± 15%) and adropin (127 ± 106%) increased (*p* ≤ .0.05) only after DSW. Significant (*p* ≤  0.01) partial correlations were found between the pre- to post-DSW changes in C1q, apelin or adropin and changes in outcome measures [e.g., C1q and MVIC (r = − 0.837), apelin and SBP (r = − 0.854), and andropin and OGTT (r = − 0.729)]. These results showed that greater decreases in plasma C1q and greater increases in apelin and adropin concentrations were associated with greater improvements in outcome measures after DSW than after ASW.

## Introduction

It is recommended that individuals engage in 150 to 300 min of moderate intensity physical activity, 75 to 150 min of vigorous-intensity physical activity, or an equivalent combination of both moderate and vigorous activities each week and muscle strengthening activities at least two days each week^1,2^. However, only 22% of adults meet these recommendations, and the number decreases with increasing age^3^. Physical inactivity is a cause of many chronic diseases^4^, and regular exercise is key for increasing health span, quality of life and well-being^5^. Thus, exercises that more efficient and effective in achieving these outcomes should be implemented, and it appears that muscle contraction type is a factor affecting these outcomes.

A previous study showed that resistance training emphasising eccentric (lengthening) contractions of the knee extensors once a week for 12 weeks increased knee extension muscle strength, functional physical fitness such as the ability to stand up and sit down in a chair as quickly as possible for 30 s, insulin sensitivity and blood lipid profiles in older men greater than training emphasising concentric (shortening) contractions^6^. Another study found that descending stair walking (DSW), in which the knee extensors predominantly undergo eccentric contractions, twice a week for 12 weeks was also more effective in improving muscle strength, functional physical fitness, blood pressure, insulin sensitivity and blood lipid profiles in older obese women than ascending stair walking (ASW), in which the knee extensors mainly undergo concentric contractions^7^. However, it is not known why exercise training involving eccentric contractions (i.e., eccentric training) produces greater adaptations of not only the muscles that are stimulated during the training but also other organs in the body to improve blood pressure, insulin sensitivity and blood lipid profiles^6,8^. It may be that changes in some biomarkers induced by exercise training involving eccentric contractions (e.g., DSW) and that involving concentric contractions (e.g., ASW) are different.

Some studies have shown that the expression of complement component 1q (C1q), which is a protein complex involved in the innate immune system^9^, increases with age and leads to muscle fibrosis and atrophy^10,11^. Previous studies^12,13^ have reported that C1q expression in the blood is a marker of sarcopenia and that the higher the concentration of C1q is in the blood, the greater the extent of sarcopenia. Watanabe et al.^13^ reported that 12 weeks of resistance training of the knee flexors and extensors three times a week with a load of 70% one-repetition maximal (1-RM) consisting of 3 sets of 10 repetitions per session decreased the serum C1q concentration by 39%, and increased maximal voluntary isometric contraction (MVIC) strength by 8–12% and muscle cross-sectional area by 7% in older adults. Thus, it may be that eccentric exercise training decreases the C1q concentration in the blood more than concentric exercise training.

Apelin is produced and secreted by adipose tissue^14^ and affects various physiological processes, including modulating cardiovascular function and glucose homeostasis, promoting insulin sensitivity and modulating energy metabolism, lipogenesis and lipolysis^15^. Kadoglou and colleagues^16^ found significant increases in the serum apelin concentration (+ 39%) after 12 weeks of aerobic exercise training in patients with type 2 diabetes mellitus (T2DM). Afshounpour and colleagues^17^ also showed that the plasma apelin concentration increased by 29% and that this increase was accompanied by improved insulin resistance after 8 weeks of aerobic and whole-body resistance exercise training in patients with T2DM. It is possible that eccentric exercise training increases the apelin concentration in the blood more than concentric exercise training.

Adropin is known as a regulator of endothelial nitric oxide (NO) synthase and NO release, and the circulating adropin concentration decreases with age^18,19^. Fujie and colleagues^19^ reported that the serum adropin concentration increased by 80% in middle-aged and older individuals after 8 weeks of aerobic cycling training three times per week and that the increase in the serum adropin concentration was significantly correlated with a decrease in carotid beta-stiffness and an increase in the plasma NO concentration. It might be that changes in the adropin concentration in the blood are greater after eccentric exercise training than concentric exercise training.

As mentioned above, we found that knee extensor MVIC strength (34%) and physical function (8–42%) were increased, systolic blood pressure (SBP) was decreased by 9%, and insulin sensitivity [e.g., oral glucose tolerance test (OGTT) results: − 12%] and lipid profiles [e.g., low-density lipoprotein cholesterol (LDLC) level: − 13%] were improved in elderly obese women who performed DSW twice a week for 12 weeks with a progressive increase in repetitions from two (110 steps × 2 = 220 steps) to 24 (110 steps × 24 = 2,640 steps) compared with those who performed ASW (15%, 4–22%, − 2% and − 7%, respectively)^7^. We hypothesised that the plasma C1q concentration would decrease to a greater extent and that apelin and adropin concentrations would increase to a greater extent after DSW training than after ASW training. To test this hypothesis, we used the stored blood samples from our previous study^7^ to measure C1q, apelin and adropin concentrations in stored plasma samples taken at baseline and after the 12-week DSW and ASW interventions^7^, compared the changes in concentrations between the DSW and ASW groups, and investigated the relationships between the changes in the blood markers and the changes in musculoskeletal parameters, functional physical fitness, cardiovascular function (resting heart rate (HR) and blood pressure), insulin sensitivity and lipid profiles, which had been evaluated in our previous study^7^.

## Results

### Baseline measurements

**Figure 1:**
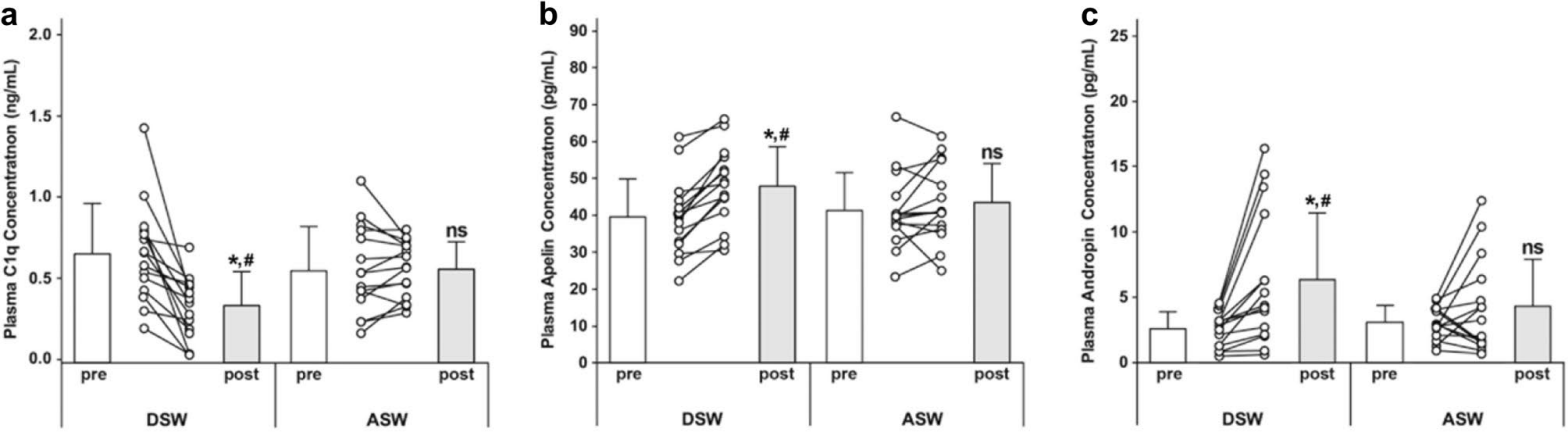
Changes (individuals, mean ± SD) in plasma complement component 1q (C1q) (**a**), apelin (**b**) and adropin (**c**) concentrations before (pre) and after (post) 12 weeks of descending (DSW) and ascending stair walking (ASW) interventions. *Significant change (*p* ≤ 0.05) compared to the baseline (pre) level in each group; ns: no significant difference (*p* ≥ 0.05) from the baseline (pre) level; ^#^Significant difference (*p* ≤ 0.05) compared to the post-intervention level in the ASW group.

No significant (*p* > 0.05) differences in any of the outcome measures (Tables 1, 2) or for plasma C1q, apelin and adropin concentrations were found between the DSW and ASW groups (Fig. 1) at the baseline.

**Table 1:** Changes (mean ± SD) between the pre- and post-training time points in the ascending (ASW; n = 15) and descending stair walking (DSW; n = 15) groups for body mass, body mass index (BMI), percent body fat, resting heart rate (HR), systolic (SBP) and diastolic blood pressure (DBP), maximal voluntary isometric contraction strength of the knee extensors (MVIC), performance in the 30-s chair stand (CS), 2-min step (2MS), 6-m walk (6 MW), 6-m tendon walk (TW) test, balance with eyes open on firm surface (EOFS), balance with eyes closed on unstable surface (ECUS), upper thigh circumference (CIR), and bone mineral density (BMD).

	Group	Pre	Post		Group	Pre	Post		Group	Pre	Post
Body mass (kg)	ASW	62.7 ± 6.4	62.1 ± 5.9^#^	BMI (kg/m^2^)	ASW	26.1 ± 0.8	25.8 ± 0.7^#^	Body fat (%)	ASW	34.6 ± 5.3	33.4 ± 4.8^#^
	DSW	62.8 ± 5.1	61.7 ± 5.2^#^		DSW	26.2 ± 1.1	25.7 ± 1.1^#^		DSW	36.4 ± 3.3	35.1 ± 3.4^#^
HR (beats/min)	ASW	80.3 ± 4.7	77.1 ± 5.1^#^	SBP (mmHg)	ASW	122.6 ± 11.7	118.5 ± 12.4^#^	DBP (mmHg)	ASW	69.3 ± 7.7	65.6 ± 7.5^#^
	DSW	79.2 ± 4.5	71.5 ± 6.2^#^^,^*		DSW	119.9 ± 9.3	109.7 ± 10.8^#^^,^*		DSW	69.1 ± 7.2	64.1 ± 7.1^#^
MVIC (N)	ASW	245.3 ± 29.4	280.5 ± 27.3^#^	CS (times)	ASW	16.5 ± 1.4	19.9 ± 1.6^#^	2MS (times)	ASW	111.0 ± 5.1	118.9 ± 6.0^#^
	DSW	262.4 ± 25.5	352.2 ± 55.7^#^^,^*		DSW	15.0 ± 1.3	21.3 ± 3.5^#^^,^*		DSW	110.1 ± 4.7	122.2 ± 14.5^#^
6 MW (m)	ASW	467.0 ± 38.9	482.6 ± 34.2^#^	TW (s)	ASW	27.1 ± 4.2	24.0 ± 2.7^#^	EOFS (SI)	ASW	0.69 ± 0.06	0.64 ± 0.07^#^
	DSW	460.1 ± 31.3	496.1 ± 41.6^#^		DSW	26.3 ± 4.0	21.6 ± 4.7^#^		DSW	0.68 ± 0.05	0.52 ± 0.09^#^^,^*
ECUS (SI)	ASW	2.86 ± 0.20	2.72 ± 0.31^#^	CIR (cm)	ASW	48.1 ± 3.7	48.4 ± 3.6^#^	BMD (m/s)	ASW	1492.3 ± 66.3	1496.8 ± 36.1
	DSW	2.88 ± 0.25	2.08 ± 0.43^#^^,^*		DSW	48.6 ± 2.6	49.2 ± 2.5^#^		DSW	1487.5 ± 55.3	1576.9 ± 69.7^#^^,^*

^#^: significant (*p* ≤ 0.05) difference compared with the pre-training level. *: significant (*p* ≤ 0.05) difference compared with the ASW group.

**Table 2:** Changes (mean ± SD) in resting glucose (GLU) and insulin (INS) concentrations, the homeostasis model assessment (HOMA) index, whole blood glycosylated hemoglobin (HbA1c) concentration, oral glucose tolerance test (OGTT) results, total cholesterol (TC), triacylglycerol (TG), low- (LDLC) and high-density lipoprotein cholesterols (HDLC) concentrations before (pre) and after (post) ascending (ASW; n = 15) and descending stair walking (DSW; n = 15).

	Group	pre	post		Group	pre	post		Group	pre	post
GLU (mg/dL)	ASW	111.5 ± 6.9	106.4 ± 6.6^#^	INS (pmol/L)	ASW	83.3 ± 6.9	79.1 ± 5.6^#^	HOMA (A.U.)	ASW	2.64 ± 0.32	2.39 ± 0.24^#^
	DSW	113.9 ± 15.5	104.2 ± 18.4^#^^,^*		DSW	85.4 ± 7.1	69.2 ± 5.3^#^^,^*		DSW	2.74 ± 0.32	2.02 ± 0.28^#^^,^*
HbA1c (%)	ASW	5.86 ± 0.35	5.88 ± 0.65	OGTT (mg/dL/2 h)	ASW	816.4 ± 69.6	798.6 ± 86.1^#^	TG (md/dL)	ASW	105.9 ± 8.6	97.9 ± 11.3^#^
	DSW	5.87 ± 0.27	5.58 ± 0.38^#^^,^*		DSW	833.7 ± 61.8	733.6 ± 80.5^#^^,^*		DSW	109.3 ± 8.4	88.1 ± 19.6^#^^,^*
TC (md/dL)	ASW	208.5 ± 18.5	201.1 ± 14.8^#^	LDLC (md/dL)	ASW	125.9 ± 12.5	116.0 ± 7.9^#^	HDLC (md/dL)	ASW	64.4 ± 6.1	64.2 ± 5.9
	DSW	215.2 ± 20.3	194.5 ± 24.9^#^		DSW	127.5 ± 11.9	110.5 ± 13.3^#^^,^*		DSW	62.1 ± 5.7	68.3 ± 7.4^#^^,^*

^#^: significant (*p* ≤ 0.05) difference compared with the pre-training level. *: significant (*p* ≤ 0.05) difference compared with the ASW group.

### Changes in outcome measures

Significant differences in the changes in resting heart rate (HR) and SBP were found between the DSW and ASW groups, with the DSW group showing a greater decrease than the ASW group (HR: interaction effect: *p* = 0.003, η^2^ = 0.480; SBP: *p* = 0.018, η^2^ = 0.341; Table 1). Significantly (*p* ≤ 0.05) greater improvements in the MVIC strength of the knee extensors (*p* < 0.001, η^2^ = 0.653), upper thigh circumference (CIR; *p* = 0.024, η^2^ = 0.313), and calcaneal bone mineral density (BMD) of the right heel (*p* = 0.002, η^2^ = 0.499) were also evident after DSW training than after ASW training (Table 1). Significant (*p* ≤ 0.05) improvements in performance in all functional physical fitness tests were observed in both the DSW and ASW groups following the 12-week training, but the magnitude of the improvements in performance in the 30-s chair stand (CS) test *p* = 0.007, η^2^ = 0.420), balance with eyes closed on an unstable surface (ECUS; *p* < 0.001, η^2^ = 0.742) and balance with eyes open on a firm surface (EOFS; *p* < 0.001, η^2^ = 0.585) was greater (*p* ≤ 0.05) after DSW training than after ASW training, as shown in Table 1. As shown in Table 2, significantly (*p* ≤ 0.05) greater changes in all insulin sensitivity (e.g., glucose concentration: *p* = 0.042, η^2^ = 0.263) and lipid profile (e.g., triglyceride (TG) concentration: *p* = 0.013, η^2^ = 0.365) parameters were found after DSW training than after ASW training.

### Changes in plasma C1q, apelin, and adropin concentrations

After 12-week DSW training, the plasma C1q concentration decreased [*p* < 0.001, eta-squared values (η^2^) = 0.599], and the apelin (*p* < 0.001, η^2^ = 0.767) and adropin (*p* = 0.003, η^2^ = 0.480) concentrations increased, but no significant changes were found after ASW training (C1q concentration: *p* = 0.885, η^2^ = 0.002; apelin concentration: *p* = 0.267, η^2^ = 0.087; adropin concentration: *p* = 0.165, η^2^ = 0.133) (Fig. 1). The magnitude of the decrease in C1q concentration (*p* < 0.001, η^2^ < 0.600) and the increase in apelin (*p* = 0.011, η^2^ = 0.378) and adropin (*p* = 0.014, η^2^ = 0.361) concentrations between the pre- to post-training time points was greater in the DSW group than in the ASW group (Fig. 1).

### Correlations between changes in C1q, apelin or adropin concentrations and changes in outcome measures

**Figure 2:**
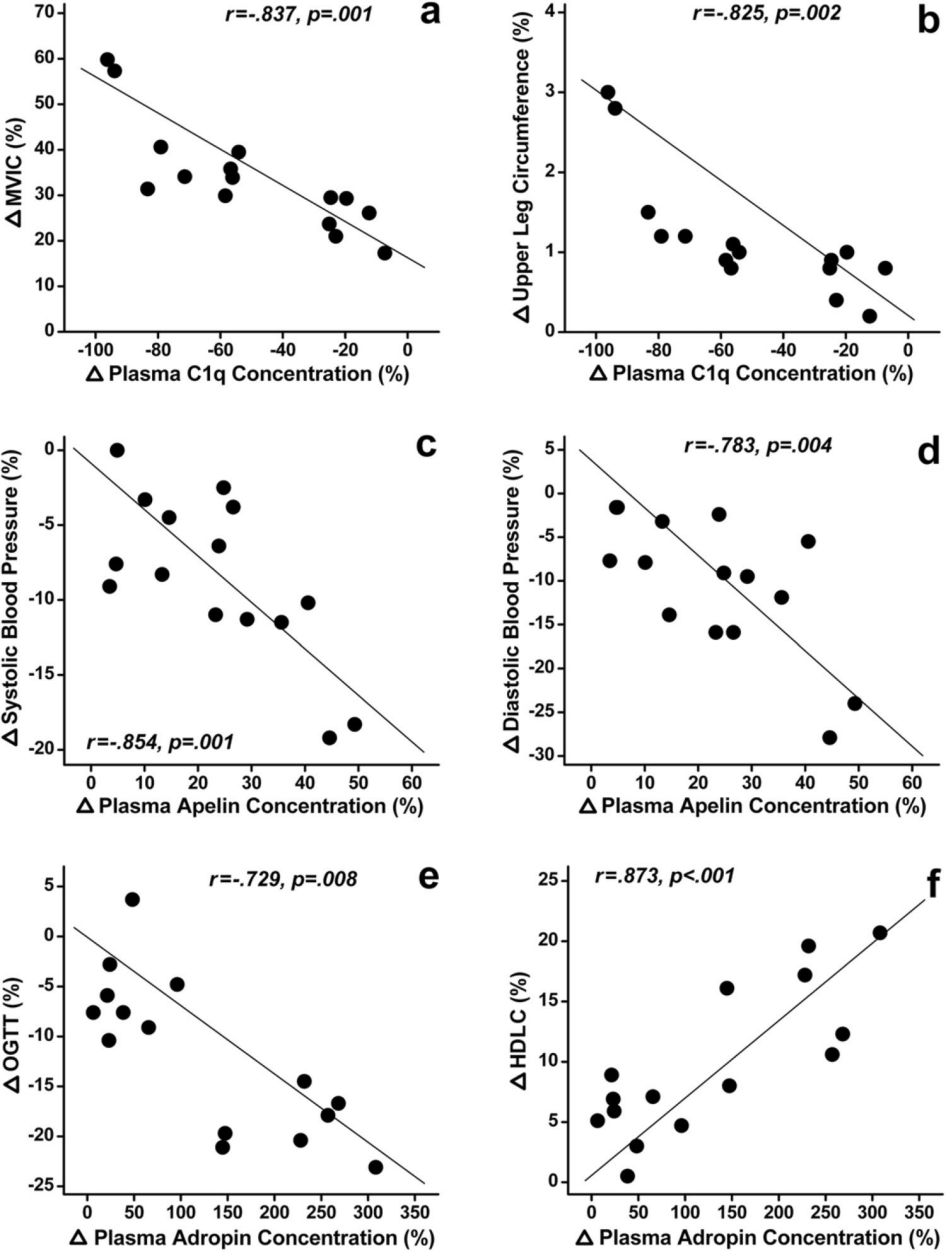
Scatter plot graphs for some selected strong correlations in Table 1. Partial correlations between complement component 1q (C1q) concentration and the maximal voluntary isometric contraction strength of the knee extensors (∆MVIC) (**a**), upper thigh circumference (∆CIR) (**b**), apelin concentration and systolic blood pressure (∆SBP) (**c**), diastolic blood pressure (∆DBP) (**d**), adropin concentration and oral glucose tolerance test (∆OGTT) results (**e**), and high-density lipoprotein cholesterols concentration (∆HDLC) (**f**) for the participants in the descending stair walking (DSW) group (n = 15).

Table 3 shows the results of partial correlation analyses of the magnitude of changes in plasma C1q, apelin, and adropin concentrations and the changes in HR, blood pressure, muscle strength, functional physical fitness, muscle and bone, insulin sensitivity, and lipid profile parameters from baseline to the post-training time point for participants in the DSW group. The normalised change in C1q concentration was significantly (*p* ≤ 0.01) correlated with the changes in MVIC (Fig. 2a), upper thigh circumference (Fig. 2b), 6-m walk (6 MW) test results and calcaneal BMD between the pre- to post-training time point in the DSW group. The normalised change in apelin concentration was significantly (*p* ≤ 0.01) correlated with the changes in SBP (Fig. 2c), diastolic blood pressure (DBP) (Fig. 2d) and 6 MW test results between pre- to post-training time points in the DSW group. The normalised change in adropin concentration was also significantly (*p* ≤ 0.01) correlated with the changes in balance with EOFS, serum glucose concentration, homeostasis model assessment (HOMA) index, OGTT results (Fig. 2e), total cholesterol (TC) concentration and high-density lipoprotein cholesterol (HDLC) concentration (Fig. 2f). In the ASW group, a significant (*p* ≤ 0.01) correlation was between changes in the apelin concentration and changes in DBP (r = − 0.732, *p* = 0.008), between changes in the apelin concentration and changes in CS (r = − 0.717, *p* = 0.010), and between changes in the apelin concentration and changes in glycosylated haemoglobin (HbA1c) concentration (r = 0.762, *p* = 0.005) only.

**Table 3:** Multiple partial correlations (partial r and p values) between the normalised changes in plasma complement component 1q (C1q), apelin and adropin concentrations between before and after 12 weeks of descending stair walking (DSW) training and the normalised changes in resting heart rate (HR), systolic (SBP) and diastolic blood pressure (DBP), the maximal voluntary isometric contraction strength of the knee extensors (MVIC), 30-s chair-stand (CS), 2-min step (2MS), 6-m walk (6 MW), 6-m tendon walk (TW) test performance, balance with eyes open on firm surface (EOFS) and eyes closed on unstable surface (ECUS), upper thigh circumference (CIR), and bone mineral density (BMD) [upper table], and resting glucose (GLU) and insulin (INS) levels, the homeostasis model assessment (HOMA) index, whole blood glycosylated hemoglobin (HbA1c) concentration, oral glucose tolerance test (OGTT) results, total cholesterol (TC) concentration, triacylglycerol (TG) concentration, low- (LDLC) and high-density lipoprotein cholesterols (HDLC) concentrations [lower table] in the participants in the descending stair walking (DSW) group (n = 15).

		HR	SBP	DBP	MVIC	CS	2MS	6 MW	TW	EOFS	ECUS	CIR	BMD
C1q	r	.446	.614	.355	− .837*	**− **.608	**− **.391	− .873*	**− **.233	.703	.523	− .825*	− .929*
	p	.098	.029	.157	**.001**	.031	.132	** < .001**	.259	.012	.061	**.002**	** < .001**
Apelin	r	**− **.573	− .854*	− .783*	.565	.594	.211	.750*	.279	**− **.407	**− **.696	.397	.591
	p	.042	**.001**	**.004**	.044	.035	.279	**.006**	.217	.122	.013	.128	.036
Adropin	r	**− **.122	**− **.348	**− **.044	.413	.378	.216	.399	.025	− .736*	**− **.578	.435	.526
	p	.368	.162	.452	.118	.141	.275	.127	.472	**.008**	.040	.104	.059

		GLU	INS	HOMA	HbA1c	OGTT	TG	TC	LDLC	HDLC			
C1q	r	.332	.322	.416	.391	.447	.035	.417	.179	**− **.630			
	p	.174	.182	.116	.132	.097	.462	.115	.310	.025			
Apelin	r	**− **.185	**− − **.483	**− **.406	**− **.360	**− **.426	**− **.316	**− **.446	**− **.119	.590			
	p	.304	.079	.122	.153	.110	.187	.098	.371	.036			
Adropin	r	− .723*	**− **.447	− .713*	**− **.471	− .729*	**− **.493	− .863*	**− **.672	.873*			
	p	**.009**	.098	**.010**	.085	**.008**	.074	**.001**	.017	** < .001**			

*: significant (*p* ≤ 0.01) correlation.

## Discussion

The main findings of the present study were that (1) changes in plasma C1q, apelin and adropin contractions were greater after DSW training than after ASW training (Fig. 1); (2) the magnitude of decrease in the plasma C1q concentration after DSW was significantly correlated with the changes in the upper thigh circumference (r = − 0.825, *p* = 0.002), BMD (r = − 0.929, *p* < 0.001), MVIC strength (r = − 0.837, *p* = 0.001) and 6 MW test results (r = − 0.873, *p* < 0.001); 3) the magnitude of increase in the plasma apelin concentration after DSW was significantly correlated with the changes in SBP (r = − 0.854, *p* = 0.001), DBP (r = − 0.783, *p* = 0.004) and 6 MW test results (r = 0.750, *p* = 0.006); and 4) the magnitude of changes in the plasma adropin concentration after DSW was significantly correlated with balance with EOFS (r = − 0.736, *p* = 0.008), glucose concentration (r = − 0.723, *p* = 0.009), HOMA index (r = − 0.713, *p* = 0.010), OGTT results (r = − 0.729, *p* = 0.008), TC concentration (r = − 0.863, *p* = 0.001) and HDLC concentration (r = 0.873, *p* < 0.001) (Table 3, Fig. 2). These results support the hypothesis and suggest that the greater training effects of DSW than ASW were associated with greater changes in plasma C1q, apelin and adropin concentrations between the baseline and post-training time points.

Watanabe and colleagues^13^ reported a 39% decrease in the serum C1q concentration in older adults after 12 weeks of bilateral resistance training of the knee flexors and extensors three times a week, which increased the MVIC strength of the muscles by 8–12% and the muscle cross-sectional area by 7%. The researchers stated that C1q was a novel biomarker of sarcopenia^13^, and a potential role for C1q concentration in the development of collagen in bones has been reported^20^. In the present study, 12 weeks of DSW training decreased the plasma C1q concentration by 51% (Fig. 1a) and increased MVIC strength and BMD by 34% and 6%, respectively. It is interesting that the magnitude of decrease in the C1q concentration in the blood and increase in muscle strength and BMD seemed to be greater after DSW than after resistance training in the aforementioned study^13^, in which the intensity of eccentric contractions performed in the resistance training protocol was likely to be higher than that in the DSW training protocol. However, the total number of eccentric contractions performed by each leg during the training period was much greater in the DSW training protocol than in the resistance training protocol. It may be that not only the intensity but also the number of eccentric contractions causes the decrease in the C1q concentration in the blood after DSW training. Since the same number of concentric contractions performed in the ASW training protocol did not decrease the plasma C1q concentration, it seems likely that eccentric contractions were responsible for the decrease in the plasma C1q concentration in the present study.

The magnitude of the increase in the plasma apelin concentration after DSW was 23% (Fig. 1b). A previous study reported a significant decrease in the apelin concentration (− 30%) after 8 weeks of aerobic exercise training [50–70% of the maximal heart rate (HR_max_)] in obese adult women^21^; however, other studies^16,17,22^ have shown significant increases in apelin concentration after aerobic exercise training. For example, Fujie and colleagues^22^ reported a 117% increase in the plasma apelin concentration after aerobic exercise training at 60–70% of the VO_2_ peak three times a week for 8 weeks in middle-aged and older adults. Kadoglou and colleagues^16^ found a 39% increase in the serum apelin concentration after 12 weeks of aerobic exercise training four times a week for 45–60 min per session in patients with T2DM. Afshounpour and colleagues^17^ also reported that a combination of aerobic (50–75% of the maximal heart rate) and whole-body resistance exercise training (30–75% of 1-RM) increased the plasma apelin concentration by 29% in patients with T2DM. Thus, the magnitude of the increase in the plasma apelin concentration observed in the present study after DSW was comparable to that reported by Afshounpour and colleagues^17^. It appears that metabolic demand during exercise is a factor affecting the increase in the apelin concentration in the blood. However, no increase in the plasma apelin concentration was evident after ASW, during which the HR was higher (114 bpm: ~ 74% of the HR_max_) than that during DSW (89 bpm: ~ 58% of the HR_max_). It may be that the intensity of ASW was too low to increase the plasma apelin concentration. It seems possible that eccentric contractions contribute to the increase in apelin concentration in the blood, probably through a different mechanism than aerobic factors. Apelin is involved in the regulation of eNOS gene expression and contributes to NO production in the endothelial cells of the aorta^23,24^. It would be interesting to investigate changes in NO concentration after DSW in comparison to after ASW or other modes of eccentric exercises.

Regarding the plasma adropin concentration, the magnitude of increase was 127% after DSW, but the increase was not significant after ASW (Fig. 1c). It appears that the extent of the increase after the 12-week DSW intervention was greater than that reported in previous studies^19,25^. Fujie and colleagues^19^ showed an 80% increase in the serum adropin concentration after 8 weeks of aerobic cycling training in middle-aged and older individuals. In contrast, two previous studies reported no significant increases in serum adropin concentrations after acute^26,27^ and chronic aerobic exercise in healthy young men and middle-aged individuals, respectively. Zhang and colleagues^25^ stated that serum adropin might underlie the improvement in endothelial function induced by aerobic exercise. It should be noted that the aerobic component of DSW was minimal, as indicated by the low HR of the participants during DSW (~ 58% of the HR_max_); however, the plasma adropin concentration more than doubled after DSW intervention, and no such increase was observed after ASW intervention (Fig. 1c). It is possible that eccentric contractions have a synergistic effect with aerobic exercise in increasing the adropin concentration in the blood after exercise training.

The magnitude of the decrease in the plasma C1q concentration and increase in the plasma apelin and adropin concentrations after DSW was strongly (r > 0.7 and *p* ≤ 0.01) correlated with the changes in some of the outcome measures (Table 3). A significant (*p* ≤ 0.01) correlation was found only between the magnitude of change in apelin concentration and DBP (r = − 0.732), CS (r = − 0.717), and HbA1c levels (r = 0.762) after ASW. After DSW, the C1q concentration changes were more strongly correlated with muscle and bone markers, the apelin concentration changes were more strongly associated with blood pressure, and the adropin concentration changes were related to insulin sensitivity and blood lipid profiles (Table 3).

The magnitude of the decrease in the plasma C1q concentration after DSW was strongly (r > 0.7 and *p* ≤ 0.01) correlated with the increase in MVIC strength, upper thigh circumference, BMD and 6 MW test results. It appears that skeletal muscle adaptations were associated with a decrease in the C1q concentration. Previous studies^12,13^ have reported that C1q expression in the blood is a marker of sarcopenia and that the greater the concentration of C1q is in the blood, the greater the extent of sarcopenia. Watanabe and colleagues^13^ found that the decrease in the serum C1q concentration after 12 weeks of resistance training was significantly correlated with the increase in muscle cross sectional area in older adults and stated that the serum C1q concentration reflected the loss of muscle mass and strength upon ageing and responded to progressive resistance training. It is known that resistance exercise elevates protein synthesis in muscles, activates satellite cells, promotes the cell cycle, and increases anabolic action^28,29^. It is possible that DSW induced these changes and increased MVIC and muscle mass along with decreasing the plasma C1q concentration. Regarding apelin concentration, the magnitude of the increase in the plasma apelin concentration was highly (r > 0.7 and *p* ≤ 0.01) correlated with the decreases in resting SBP and DBP as well as 6 MW test results. Apelin is involved in many physiological processes, including the regulation of blood pressure and cardiac function^15,29^. Pang and colleagues^30^ reported that apelin administration reduced the systolic pressure of hypertensive rats, and Ishida and colleagues^23^ showed that the hypotensive effect of apelin was mediated through the Akt/eNOS pathway. Andersen and colleagues^31^ stated that apelin modulated eNOS expression, induced eNOS-dependent vasodilatation in the systemic and pulmonary circulation, and counteracted angiotensin-II-induced vasoconstriction. It has been documented that vasodilation mediated by NO in contracting muscles via increases in NO concentrations decreases blood pressure^32^. It should be noted that the plasma apelin concentration did not change after ASW training; however, the HR and SBP were also decreased significantly after ASW training, although the magnitude of decrease was less than that after DSW training. It seems possible that apelin was not the only factor contributing to the improvements in HR and SBP induced by DSW training.

Regarding adropin concentration, the magnitude of changes in the plasma adropin concentration was strongly (r > 0.7 and *p* ≤ 0.01) correlated with balance with EOFS, insulin sensitivity (glucose concentration, HOMA index, OGTT results), and lipid profiles (TC and HDLC concentrations), as shown in Table 3. It is possible that adropin improved dyslipidaemia, glucose tolerance and insulin resistance through regulation of NO bioavailability^33,34^. Shahjouei and colleagues^35^ showed that adropin promoted glucose oxidation by decreasing the acetylation of pyruvate dehydrogenase complex (PDHC), which is a rate-limiting factor for glucose oxidation, and downregulated the expression pyruvate dehydrogenase kinase-4 (PDK-4), which is a PDHC inhibitor. Several studies have demonstrated the roles of adropin in regulating glucose metabolism^36–41^. Adropin has also been shown to regulate fuel selection in skeletal muscle to reduce fat oxidation while enhancing glucose oxidation^42,43^. It may be that the descending stair walking intervention effects on improving insulin resistance, blood lipid profile, and cardiovascular health were related to increased blood flow and capillary density associated with the increased adropin^33^. It is known that peroxisome proliferator-activated receptor gamma-activating factor 1 alpha (PGC-1α) affects the activity of carnitine palmitoyltransferase-1 (CPT-1) and PDK-4, which play key roles in the oxidation of fatty acids and glucose oxidation via the PDK pathway^44,45^. Ghoshal and colleagues^46^ reported that the plasma adropin concentration was inversely related to the plasma LDLC concentration in humans, suggesting a link with hepatic lipid metabolism. Thus, the large increase in adropin concentration after DSW may suggest that PGC-1α and its downstream pathways were more activated by DSW than ASW. It has also been documented that circulating adropin promotes the production of NO and increases NO bioavailability, which improves arterial stiffness after aerobic exercise training^22^. However, no significant partial correlations were found between the increase in adropin concentration and the decrease in SBP in the present study (Table 3).

The mechanisms underlying the greater changes in plasma C1q, apelin and adropin concentrations after DSW training than after ASW training are not clear, but some speculations can be made. The main difference between DSW and ASW was the type of muscle contraction performed by the knee extensors; eccentric contractions were mainly performed during DSW, and concentric contractions were performed during ASW^7^. It has been shown that eccentric contractions promote the anabolic signalling pathway (elevating protein synthesis in muscle, increasing the satellite cell number, and promoting the cell cycle) to a greater extent than concentric contractions^47,48^. It should be noted that the intensity of eccentric contractions performed during DSW was lower than the maximal eccentric contraction strength. Samuel and colleagues^49^ examined biomechanical functional demand placed on the knee and hip muscles of older adults during DSW and ASW and reported that the functional demand relative to the MVIC strength of the knee extensors was greater during DSW (73%) than ASW (42%). D’Lima and colleagues^50^ reported that the peak force of the knee extensors was 2.8 × body weight (BW) during walking, 2.8 × BW during ASW, 3.1 × BW during DSW, and 3.6 × BW during jogging. Eccentric loading during normal walking is approximately 25% of the MVIC strength of the knee extensors^51^. Thus, the actual loading to the knee extensors during DSW and ASW does not appear to be largely different, but it seems possible that the type of muscle contraction of the knee extensors itself (eccentric vs concentric) rather than the force was the key factor contributing to the different changes in C1q, apelin and adropin concentrations between the DSW group and ASW group.

Myokines are cytokines and other peptides that are secreted from skeletal muscles, especially in response to exercise; these peptides function like hormones either locally within the muscle or by targeting distant organs^52^. Apelin is classified as a myokine since it is produced and secreted not only by adipose tissue but also by skeletal muscle in response to exercise^53^. Adropin is considered a neuropeptide since energy homeostasis-associated (ENHO) genes are abundant in the brain, including the ventromedial and lateral hypothalamic nuclei, which regulate appetite and autonomic function^54^. Hepatic ENHO expression is associated with the expression of genes involved in glucose and lipid metabolism^54^. The present study revealed that apelin and adropin concentrations increased only after DSW, during which eccentric contractions were mainly performed. Eccentric contractions can produce high levels of mechanical tension per active motor unit, stretch-induced strain, and a greater propensity for muscle damage than concentric contractions^55^. It may be that eccentric contractions triggered the secretion of apelin and adropin by imposing greater mechanical stresses on muscle fibres, surrounding connective tissue, and capillaries and/or blood vessels. Further studies are needed to compare the effects of eccentric and concentric contractions on the secretion of myokines or neuropeptides.

Ageing is unavoidable, but decreases in physical function upon ageing can be attenuated by performing regular exercise, which is easier said than done. As the aged population increases worldwide, increasing health span is critical, and increasing physical activity and exercise is crucial. One of the barriers to physical activity is a lack of confidence in one’s ability to be physically active (low self-efficacy)^56^. As shown in the present study, the beneficial effects of DSW are encouraging to some people who are less fit and less confident in performing more physically demanding exercises. The significance of eccentric contractions in exercise prescription and the use of eccentric exercises such as DSW, downhill walking, or sitting to a chair slowly should be more strongly emphasised.

The present study had several limitations. First, the participants of the study were obese elderly women; thus, the results of the study may not generalize to young, adult and middle-aged men and women. Second, only plasma C1q, apelin and adropin concentrations were measured in the present study, and other parameters, such as well-known exercise-related biomarkers (blood adiponectin and leptin concentrations)^57^, were not evaluated in the present study. Third, the total number of participants in each group was 15, which was not appropriate for multiple regression analysis. It should also be noted that we did not control for the daily physical activity of the subjects during the experimentation, although we instructed and reminded the participants to maintain their normal daily lifestyles. Last, the present study was rather descriptive, and a mechanistic investigation of the factors affecting the changes in C1q, apelin and adropin concentrations following DSW was absent. Future studies considering these limitations and examining whether myokine and other biomarker responses are different following eccentric and concentric exercise are warranted.

In conclusion, the present study showed that the magnitude of changes in plasma C1q, apelin and adropin concentrations between pre- to post-training time points was greater in the DSW group than in the ASW group. It appears that the decrease in the plasma C1q concentration was more strongly associated with the increase in muscle and physical functions, that the increases in plasma apelin and adropin concentrations were more strongly associated with improvements in insulin sensitivity and lipid profiles, and that the increase in the plasma apelin concentration was also associated with decreases in the resting HR and blood pressure as well as musculoskeletal and fitness. Further studies are required to investigate how greater changes in C1q, apelin and adropin concentration are induced by DSW intervention than by ASW intervention using a larger sample size.

## Methods

### Participants

We conducted secondary analyses of muscular, physical fitness, bone, cardiovascular, insulin sensitivity and lipid profile data from the previous intervention study in which older obese women performed descending (DSW) or ascending stair walking (ASW) training twice a week for 12 weeks. Thirty obese (body fat ≥ 30%) older (60–82 years old) women who had been sedentary provided informed consent to participate in the study, which was approved by the Joint Institutional Review Board of Taipei Medical University (approval #: 201502034). The participants were recruited from several Taipei City District health centres (Heihu, Shilin, Beitou). Their eligibility and suitability to participate in the study was determined by the International Physical Activity Questionnaire (IPAQ) and a health and medical questionnaire. An experienced orthopaedist checked the participant’s lower extremities to ensure that the participants had no knee or ankle problems, degenerative joint and muscle disease, or osteoarthritis that might be exacerbated by stair walking exercise. The participants were quasi-randomly divided into the DSW and ASW intervention groups (n = 15 per group) so that the average baseline MVIC strength of the knee extensors, age, body mass, and percent body fat were similar between the groups^7^. Briefly, a maximum of eight participants were recruited at the same time, and they were ranked based on the MVIC strength, placed into one of the two groups by a lottery from the top pair to the bottom pair, to ensure that the baseline of MVIC strength was similar between the DSW and ASW groups. The sample size was estimated on the basis of an effect size of 1.0, an α-level of 0.05, and a power (1-β) of 0.80 for the possible difference in the increase in the MVIC strength of the knee extensors between the groups based on our pilot study using G*Power (G*Power 3.1.9.2; Heinrich-Heine-Universitat Dusseldorf, Dusseldorf, Germany). According to the analysis, 14 participants per group were necessary. Considering the possibility of participant dropout, 15 participants per group were recruited. No significant differences in any of the physiological characteristics were observed between the DSW (mean ± standard deviation (SD): age, 65.4 ± 6.6 y; height, 154.9 ± 5.5 cm; body mass, 62.8 ± 5.1 kg; body mass index (BMI), 26.2 ± 1.1; percent body fat, 36.4 ± 3.3%) and ASW (age, 67.5 ± 7.1 y; height, 155.6 ± 6.2 cm; body mass, 62.7 ± 6.4 kg; BMI, 26.1 ± 0.8; percent body fat, 34.6 ± 5.3%) groups at baseline.

### Exercise intervention

The exercise intervention was described in a previous study^7^. Briefly, all participants performed progressive exercise training on the stairs of a 10-story building twice a week for 12 weeks^7^. An elevator was used to take the participants in the DSW group from the first to sixth floor and to take those in the ASW group from the sixth to first floor. Thus, the DSW group did not walk up stairs, and the ASW group did not walk down stairs during the exercise intervention. The number of stairs from the first to the sixth floor was 110 (five floors, 22 stairs per floor). The walking tempo was approximately 1 s per step; thus, to descend or ascend 110 stairs, it took approximately 2 min. The exercise volume was gradually increased for both groups over 12 weeks by increasing the number of repetitions by two every week (i.e., first week, 2 repetitions per session; 12th week, 24 repetitions per session)^7^. It took approximately 5 min to perform two repetitions, including the time waiting for and riding on the elevator, and the time taken to perform 24 repetitions was approximately 60 min, including approximately 12 min waiting for the elevator or in the elevator. Thus, the intervention was an intermittent exercise during which an approximately 2-min walk followed by a 30-s rest was repeated.

During stair walking, HR was recorded with a Polar RS800CXN monitor (Polar Electro, Kempele, Finland), and SBP and DBP were measured using an automated sphygmomanometer (MS 150f., Rossmax International Ltd., Taipei, Taiwan) before, during (HR only) and immediately after each session^7,58^.

### Outcome measures

The participants were familiarised with the testing procedures a week before the first exercise session. The details of the familiarisation session and criterion measures were reported in a study by Chen and colleagues^7^. Briefly, the criterion measures were height, body mass, percent body fat, resting HR, SBP, DBP, upper thigh circumference; right calcaneus bone mineral density (BMD), MVIC strength of the right knee extensors, performance in functional fitness tests for senior adults, including the 30-s chair stand (CS), 2-min step (2MS), 6-m walk (6 MW) and 6-m tandem walk (TW), and balance (balance with EOFS and ECUS) tests; levels of insulin sensitivity markers (resting glucose and insulin concentrations, HOMA index, HbA1c concentration, OGTT results) and blood lipid profiles (TG, TC, HDLC, and LDLC concentrations)^7,59^.

The participants rested in the supine position for at least 10 min before measurement of HR, SBP, and DBP, and the measurements were taken while the participants were lying supine on a padded table. The calcaneal BMD of the right heel of each participant was assessed using a bone sonometer (CM-100; Furuno Electric Co., Hyogo, Japan). Speed of sound (m/s) was used as a quantitative ultrasound parameter determined by the width of the heel and time delay between initial transmission and subsequent receipt of sound waves^7,60^.

Assessment of MVIC strength of the knee extensors^56,60–62^; functional physical fitness tests for senior adults, i.e., the CS, 2MS, 6 MW, TW; and two balance tests (balance with EOFS and ECUS), which were carried out using a Biodex BioSway Portable Balance System (Model 950-460; Biodex Medical Systems, Shirley, NY, USA), were performed 3 days before the first training session to obtain baseline measures and 4 days after the last training session to evaluate the effects of the training^7^. Resting HR, blood pressure, and upper thigh circumference were also measured at these time points^7^.

Approximately 10 ml of blood and another 3 ml of blood for measuring HbA1c concentration were drawn from the cubital fossa region of the arm by the same standard venipuncture technique 3 days before the first training session and 4 days after the last training session^7^. Five millilitres of venous blood (10 ml) was put in a vacutainer tube containing dipotassium ethylenediaminetetraacetic acid (Becton Dickinson and Company, Plymouth, UK), and another 5 mL of blood was left in the original vacutainer tube with a serum separator and clotting activator (silica) (Becton Dickinson and Company, Plymouth, UK) for 30 min at room temperature and centrifuged (2000 rpm) for 10 min to obtain plasma and serum, respectively. Serum samples were used for insulin sensitivity measures (i.e., resting glucose and insulin concentrations, HOMA index, HbA1c concentration) and lipid profiles (serum TG, TC, LDLC and HDLC concentrations). The remaining plasma samples were stored at − 80 °C for analyses of C1q, apelin and adropin concentrations. For the OGTT, capillary blood samples were obtained from the fingertips of each participant using sterilised Softclix lancets (Roche Diabetes Care Ltd., Surrey England, UK) before and 30, 60, 90 and 120 min after consumption of a 75-g standard glucose drink, and blood glucose concentrations were measured with an analyser (Johnson & Johnson, Livingstone, Scotland, UK). The detailed procedures used for analyses of glucose, insulin, OGTT, HbA1c, HOMA, TG, TC, LDLC, and HDLC were described previously^7,63–65^.

### C1q, apelin and adropin concentrations

Plasma C1q, apelin and adropin concentrations were measured by enzyme-linked immunosorbent assay (ELISA) using commercially available kits [C1q: Human C1q ELISA Kit, GA-E0374HM, GenAsia Biotech Co., Ltd, Shanghai, China; apelin: Human AP12 ELISA Kit, GA-E0024HM, GenAsia Biotech Co., Ltd, Shanghai, China; adropin: Human Adropin (ENHO) ELISA Kit, CSB-EL007669HU, CUSABIO®, Wuhan, China] according to the instructions of the manufacturer. The detection limits of the plasma C1q, apelin and adropin assays were 0.156–10 ng/ml, 31.25–2,000 pg/ml and 1.56–100 pg/ml, respectively. The reliability of the plasma C1q, apelin and adropin concentrations was checked by the coefficient of variation (CV) and intraclass correlation (r), which were 3.9% (r = 0.953), 9.2% (r = 0.950) and 9.7% (r = 0.900) for plasma C1q, apelin and adropin, respectively.

### Statistical analyses

The data were assessed by the Shapiro–Wilk test for normality and by the Levene test for the homogeneity of variance assumption. These tests showed that the data for all parameters were normally distributed, and the variance was assumed to be homogenous. The baseline values of all dependent variables before DSW and ASW training exercise bouts were compared by t-tests. Changes in the all measures and plasma C1q, apelin and adropin concentrations over time were compared between the DSW and ASW groups by mixed-model two-way analysis of variance (ANOVA). When a significant interaction effect was found, Tukey’s post hoc test was performed. η^2^ was calculated as a measure of effect size and was classified as follows: ~ 0.02: small effect; ~ 0.13: medium effect; and > 0.26: large effect^66^. The significance level was set at *p* ≤ 0.05 for all dependent variables. Partial correlation analyses controlling for possible confounding factors (age, height, body mass, BMI, and percent body fat) were used to assess the relationships between C1q, apelin or adropin concentrations and all other variables in the DSW and ASW groups. The significance level was set at *p* ≤ 0.01 for multiple partial correlation analyses based on the small sample size (n = 15) for each group^67^. The data are presented as the mean ± SD.

### Ethical statement

All methods in the study complied with the current guidelines, regulations and laws of Taiwan where the study was performed in accordance with the 1964 Helsinki declaration.

### Ethical approval

All experimental protocols were approved by the Joint Institutional Review Board of Taipei Medical University (approval #: 201502034).

### Informed consent document

A written informed consent was obtained from each participant of the study.

## Data Availability

All data generated or analysed during this study are included in this submitted manuscript, and the data of the current study are available from the corresponding author on reasonable request.
